# Knowledge, Attitudes, and Practices Regarding Antimicrobial Resistance and Associated Factors Among Healthcare Workers in Addis Ababa, Ethiopia: A Cross-Sectional Study

**DOI:** 10.1155/cjid/5354292

**Published:** 2025-09-26

**Authors:** Abel Getu, Tesfaye Solomon, Nathan Fikre, Elizabeth Eyasu, Yaregal Fufa, Misgana Tazebachew, Zewdie Aderaw Alemu

**Affiliations:** ^1^Public Health Emergency Management Wing, Ethiopian Public Health Institute, Addis Ababa, Ethiopia; ^2^Addis Ababa City Administration Health Bureau, Addis Ababa, Ethiopia; ^3^Individual Health Consultant, Addis Ababa, Ethiopia; ^4^School of Public Health, Department of Epidemiology, St. Paul's Hospital Millennium Medical College, Addis Ababa, Ethiopia

**Keywords:** antimicrobial resistance, attitude, Ethiopia, healthcare workers, knowledge, practices, primary healthcare

## Abstract

**Background:** Antimicrobial resistance (AMR) is a growing public health concern in Ethiopia, marked by high resistance rates among common pathogens and widespread misuse of antimicrobials, particularly among healthcare providers. This study aimed to assess the knowledge, attitudes, practices (KAP), and influencing factors related to AMR among healthcare workers (HCWs) in health centers of Addis Ababa.

**Methods:** A cross-sectional study design was conducted among 485 HCWs in Addis Ababa from July 1, 2023, to November 25, 2023. Participants were HCWs involved in antimicrobial prescribing at randomly selected health centers in three subcities. Data were collected using structured questionnaires and analyzed using SPSS Version 26. Multivariable logistic regression was performed to identify significant associations at *p* < 0.05, with adjusted odds ratios (AORs) and 95% confidence intervals (CIs) reported.

**Results:** A total of 473 HCWs participated, yielding a response rate of 97.5%. While 66.8% of HCWs demonstrated good knowledge about AMR, 61.3% exhibited unfavorable attitudes, and only 43.3% had satisfactory practice scores. Factors significantly associated with poor AMR practices included age (30–39 years; AOR = 0.29, 95% CI: 0.12–0.70), being a general practitioner (AOR = 4.26, 95% CI: 1.52–11.90), holding a degree (AOR = 0.47, 95% CI: 0.26–0.88), working in the outpatient department (AOR = 3.78, 95% CI: 1.82–7.86), lack of training (AOR = 0.16, 95% CI: 0.09–0.29), good knowledge (AOR = 0.45, 95% CI: 0.28–0.72), unfavorable attitudes (AOR = 1.79, 95% CI: 1.17–2.85), and lack of guideline consultation (AOR = 0.34, 95% CI: 0.22–0.52).

**Conclusions:** The study found that younger HCWs, general practitioners, and those working in outpatient departments were more likely to exhibit better AMR practices. Conversely, lack of training and guideline consultation negatively impacted practices. Addressing knowledge gaps, improving attitudes, and reinforcing practices through targeted interventions are essential for effective AMR management in health centers.

## 1. Introduction

Antimicrobial resistance (AMR) is a growing global public health threat, driven by microorganisms developing resistance to antimicrobial drugs. This results in increased morbidity, mortality, healthcare costs, and reduced productivity, with projections suggesting 10 million annual deaths and $3.4 trillion in lost GDP by 2050 if unmanaged [1–3]. Compounding this issue, the World Health Organization (WHO) identified only 6 of 32 antibiotics under clinical development in 2019 as innovative, leaving a critical therapeutic gap [4, 5]. Carbapenem-resistant Gram-negative bacilli, for instance, already account for approximately 2% of infections worldwide [6, 7], underscoring the urgency of addressing AMR.

To combat AMR, the WHO promotes antimicrobial stewardship (AMS) as a core pillar of health system strengthening, alongside infection prevention and control (IPC) and medicine safety. AMS optimizes antimicrobial use through strategies like the Access, Watch, Reserve classification (AWaRe), surveillance, and evidence-based prescribing, thereby reducing resistance, improving patient outcomes, and curbing unnecessary costs [8, 9].

Low- and middle-income countries face heightened AMR risks due to poverty, poor sanitation, and weak healthcare infrastructure [10]. In sub-Saharan Africa, 14.8% of infections involve vancomycin-resistant enterococci [8], while East Africa reports 50% of infections caused by extended-spectrum beta-lactamase-producing Gram-negative bacteria [11]. Despite this, AMR surveillance remains fragmented, with limited data on meningitis, pneumonia, and bloodstream infections—conditions critical to public health [12].

Ethiopia exemplifies these systemic gaps. High resistance rates are reported for pathogens like *Escherichia coli* and *Klebsiella pneumoniae* [7, 13, 14], with 57.6% of prescriptions inappropriately containing antibiotics [9]. Misuse by healthcare workers (HCWs), including incorrect dosing and empirical prescribing, is widespread, exacerbated by a lack of national policies, surveillance systems, and HCW training [6, 7, 15].

Despite HCWs' pivotal role in prescribing and dispensing antimicrobials, Ethiopia lacks comprehensive studies on their knowledge, attitudes, and practices (KAP) regarding AMR, particularly in Addis Ababa—a major healthcare hub. This study assessed HCWs' KAP, identified drivers of misuse, and proposed context-specific interventions to strengthen AMS. By addressing these gaps, the findings aim to inform strategies for mitigating AMR in Ethiopia and similar settings.

## 2. Methods

### 2.1. Study Area

The study took place in Addis Ababa, Ethiopia's capital city, home to an estimated 5.4 million people [16]. The city is connected to other major Ethiopian cities via road networks and public transportation systems. Addis Ababa is situated at an altitude of 2450 m and has a subtropical highland climate, making it an attractive destination for international travel [17]. The city's healthcare system is relatively well developed and comprises public and private health facilities, including hospitals, health centers, and clinics [18]. To combat AMR, the Ethiopian government has implemented programs such as the National AMR Containment and Prevention Strategy. These initiatives focus on enhancing knowledge, promoting appropriate antibiotic use, and strengthening IPC measures among healthcare professionals [19].

### 2.2. Study Design and Period

A facility-based cross-sectional study design was conducted among randomly selected health centers in Addis Ababa from July 1, 2023, to November 25, 2023.

### 2.3. Participant Selection

Eligible participants were HCWs who had been working in health centers for at least 6 months prior to data collection and were involved in the prescribing of antimicrobials. We excluded professionals who were not available during two consecutive visits on working days.

### 2.4. Study Variables

#### 2.4.1. Dependent Variable

• AMR practices among HCWs (good or poor)

#### 2.4.2. Independent Variables

• Sociodemographic characteristics: age, sex, education level, profession, years of experience, and primary department
• Knowledge related: safety of antibiotics, utilization of antibiotics, recommended standards, and training on AMR
• Attitude related: perceptions and beliefs of HCWs on appropriate use of antibiotics, organizational support, self-efficacy, and antimicrobial stewardship
• Practice related: availability and accessibility of guidelines and protocols on AMR at their workplace, barriers to the appropriate use of antimicrobial agents, organizational support for addressing AMR, prescription of antimicrobials, antimicrobial susceptibility testing (AST), IPC practices

### 2.5. Sample Size Determination

The study calculated sample sizes for knowledge, attitudes, and practices related to AMR using simple population proportions. The largest sample size was for knowledge. Specifically, the sample size for knowledge was based on the following assumptions: a proportion of HCWs with good knowledge (74.2%) [20], a 95% confidence interval (CI), and a 5% margin of error, resulting in a sample size of 294. Considering a design effect of 1.5 and adding a 10% nonresponse rate, the final sample size was 485.

### 2.6. Sampling Technique and Procedure

A multistage sampling technique was used in this study. In the first stage, three subcities were selected randomly out of 12 subcities in Addis Ababa. Next, health centers in each selected subcity were selected randomly and allocated proportionally based on the number of available health centers in each selected subcity. Then, the HCWs involved in the prescription of antimicrobials from each selected health center were categorized into medical doctors, nurses, and health officers. Finally, study participants were allocated proportionately among these professional categories based on the number of professionals in each category. Accordingly, 160, 155, and 158 study participants were included in this study from three subcities, namely, the Kolfe Keranio, Lideta, and Nifas Silk Lafto subcities, respectively.

### 2.7. Data Collection Tools

The data collection tool was adapted from a validated survey questionnaire developed by the WHO [1]. The interviewer administered structured questionnaires via face-to-face interviews using Google Forms, assisted by trained data collectors. Before the interviews, the data collectors explained the study objectives, obtained informed consent, and ensured that the participants understood and answered all the questions.

### 2.8. Data Quality Assurance

A pretest was conducted on 15 HCWs in Addis Ababa who were not part of the study to assess the clarity, appropriateness, and completeness of the questionnaire. The questionnaire was revised after identifying ambiguities and receiving feedback from participants. Five data collectors were recruited and received comprehensive one-day training covering the study objectives, ethical considerations, questionnaire content, and data collection procedures. They were well equipped to complete face-to-face interviews and administer the questionnaire accurately. Data collection was scheduled and coordinated with health centers to minimize disruption. The principal author conducted daily quality control and periodically supervised and observed the participants to address any issues. The collected data were rigorously checked for inconsistencies, errors, or missing values.

### 2.9. Data Processing and Analysis

The Google Forms-coded questionnaires were analyzed with SPSS version 26. Univariate analyses assessed central tendency and dispersion for continuous variables, while frequency distribution was applied to categorical data. Bivariate analysis identified candidate variables with a *p* value < 0.25, followed by multivariate analysis to pinpoint independent predictors and control for confounders. Multicollinearity was assessed using the variance inflation factor, and model adequacy was confirmed via Hosmer and Lemeshow's goodness-of-fit test. Adjusted odds ratios (AORs) and *p* values < 0.05 (with 95% CI) indicated statistical significance for variables retained in the final model.

### 2.10. Operational Definitions

#### 2.10.1. Attitude

Attitude was assessed using 13 questions. Each correct answer scored 1 point, and incorrect answers scored 0. Total scores ranged from 0 to 13 and were categorized based on the attitude mean.

• Favorable attitude: Scores above the mean (≥ 88% of total points)
• Unfavorable attitude: Scores below the mean (< 88% of total points) [9, 21].

#### 2.10.2. HCWs

They are professionals who were involved in the prescription of antimicrobials and provide medical services, including doctors, nurses, and health officers (public health) [22].

#### 2.10.3. Knowledge

Knowledge was evaluated using 9 questions. Each correct answer scored 1 point, and incorrect answers scored 0. Total scores ranged from 0 to 9 and were categorized based on the knowledge mean.

• Good knowledge: Scores above the mean (≥ 51% of total points)
• Poor knowledge: Scores below the mean (< 51% of total points) [21].

#### 2.10.4. Practice

Practices were measured using 14 questions. Each correct answer scored 1 point, and incorrect answers scored 0. Total scores ranged from 0 to 14 and were categorized based on the practice mean.

• Good practice: Scores above the mean (≥ 62% of total points)
• Poor practice: Scores below the mean (< 62% of total points) [21].

## 3. Results

### 3.1. Sociodemographic Characteristics of the Respondents

A study was conducted with 473 HCWs, yielding a response rate of 97.5%. The mean age of the participants was 30.6 years, with a standard deviation (SD) of ±5.5 and ranging from 22 to 49 years. The majority of the participants, 263 (55.6%), were nurses in their profession. The mean work experience of the respondents was 7.0 years (SD + 4.8), with the largest proportion (43.6%) having less than 5 years of experience (Table 1).

### 3.2. Respondents' Knowledge of AMR

**Figure 1:**
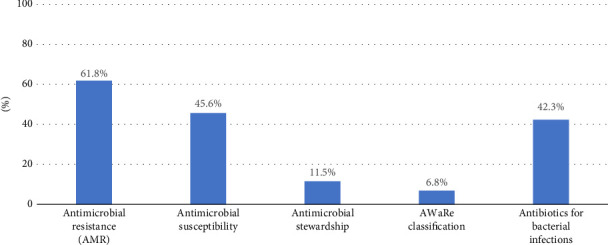
Participants' familiarity with fundamental concepts of antimicrobial resistance.

The study revealed that 66.8% (95% CI: 62.4, 71.0) of HCWs in Addis Ababa had good knowledge (Figure 1). However, a notable misconception was observed, with 42.9% of respondents believing that antibiotics could treat sore throats. Furthermore, 54.3% of the respondents mistakenly believed that antibiotics could treat viral infections, and 55.2% believed that ceftriaxone could treat methicillin-resistant *Staphylococcus aureus* (MRSA), highlighting prevalent misconceptions.

### 3.3. The Respondents' Attitudes Toward AMR

The overall favorable attitude score of HCWs regarding AMR was 61.3% (95% CI: 56.8, 65.7). A significant proportion (93.3%) acknowledged AMR as a public health problem, demonstrating high awareness and concern. However, challenges such as a perceived lack of diagnostic tools (75.6%) and gaps in regular awareness and education activities related to AMR (65.5%) were noted. Additionally, 46.1% believed that antimicrobials were overused in their facility. Furthermore, 42.9% expressed a lack of confidence in optimally prescribing antibiotics, indicating areas for improvement in prescribing practices.

### 3.4. The Practice of AMR in Responders

The overall good practice score among HCWs was 43.3% (95% CI: 38.8, 47.9), indicating that the practice level was lower than the knowledge and attitude levels. Notably, 36.2% of the HCWs administered antimicrobials without a clinically confirmed diagnosis for the patients, while 84.8% did not undergo AST before treatment initiation. Two-thirds (67.9%) of the HCWs prescribed drugs based on pharmaceutical company recommendations, yet 85.2% were not prescribed drugs upon patient request. Only 48% consulted treatment guidelines, but 62.8% educated patients on rational antimicrobial use. However, 69.8% reported no random sampling for AMR testing, indicating gaps in surveillance practices.

### 3.5. Factors Affecting Respondents' Practice of AMR

A multivariable analysis identified factors associated with AMR practices. Variables with a *p* value < 0.25 in the bivariate logistic regression were included in the multivariable model, and those with a *p* value < 0.05 were deemed significant (Table 2).

The study revealed that participants aged 30–39 years had significantly poorer practices toward AMR by 71% than did those aged 40 or older (AOR: 0.29, 95% CI: 0.12–0.70). Participants with degrees had a 53% lower likelihood of engaging in AMR practices than did those with master's degrees (AOR: 0.47, 95% CI: 0.26–0.88). Compared with nurses, general practitioners were 4.26 times more likely to practice AMR (AOR: 4.26, 95% CI: 1.52–11.90).

The study revealed that the odds of poor AMR practices were 3.78 times greater among HCWs working in the outpatient department than among those working in the inpatient department (AOR: 3.78, 95% CI: 1.82–7.86). A lack of training on AMR in the past year significantly reduced the odds of demonstrating ‘good practices' toward AMR by 84% compared to training (AOR: 0.16, 95% CI: 0.09–0.29).

Surprisingly, HCWs with ‘good knowledge' about AMR had 55% lower odds of having ‘good practice' than did those with ‘poor knowledge' (AOR: 0.45, 95% CI: 0.28–0.72). Positive attitudes toward AMR were associated with greater odds of demonstrating ‘good practices' (AOR: 1.79, 95% CI: 1.13–2.85). The study revealed that HCWs who did not adhere to any guidelines before being prescribed antimicrobials experienced a 66% decrease in their practice (AOR: 0.34, 95% CI: 0.22–0.52).

## 4. Discussion

HCWs play a vital role in the prevention and control of AMR by prescribing antimicrobials wisely, controlling the transmission of drug-resistant microbes, and promoting awareness in primary health care. Thus, this study provides insights into the knowledge, attitudes, and practices of HCWs in Addis Ababa, Ethiopia.

The study revealed that 66.8% of HCWs in Addis Ababa had a good level of knowledge about AMR concepts. This finding is similar to recent studies in Ethiopia in which 72.2% of hospital physicians and nurses [23] and 74.2% of HCWs had good knowledge of AMR [20]. However, this percentage is higher than that reported in a study conducted among paramedical staff toward AMR in Dire Dawa, Ethiopia, at 62.8% [24] and lower than that reported in a study conducted in South Africa, which revealed that 95.8% of HCWs have a high awareness of AMR [25]. This might be because the current study involved only HCWs from health centers in the capital city and did not include hospital staff.

This study revealed that 55.2% of respondents believed that ceftriaxone could treat MRSA, a misconception that poses a significant risk in the context of AMR. This misconception was also observed in a previous study conducted in the Amhara region of Ethiopia, where only a small percentage of healthcare providers had adequate knowledge about MRSA [23]. Another concerning finding was that 54.3% of the respondents believed that antibiotics could treat viral infections. This misconception contributes to inappropriate antibiotic use and the development of AMR, emphasizing the need for targeted educational interventions to rectify these misconceptions.

While a pre-post evaluation study conducted by the WHO demonstrated an increase in knowledge regarding the AWaRe classification (categorizing antibiotics into “access,” “watch,” and “reserve” groups) from 39.1% to 75.4% [26], the findings of our study revealed that only 6.8% were familiar with the AWaRe classification, and only 11.5% were acquainted with antimicrobial stewardship. This shows less coverage of program implementation and limited knowledge of the concept among HCWs, suggesting a gap in their understanding of strategies for responsible antibiotic use in this area.

This study revealed that HCWs in Addis Ababa had unfavorable attitudes (attitude score of 61.3%) toward responsible antimicrobial use. Interestingly, this finding contrasts with those of previous Ethiopian studies that reported attitudes of 83.9% among HCWs [20] and 96.3% among health professionals favoring AMR awareness [27]. This might be because all studies highlight the high level of recognition of AMR as a serious public health problem in Ethiopia, requiring a commitment among HCWs to address this challenge. Although our study's attitude level was lower, it still reflects a significant positive disposition toward addressing AMR. Effective interventions can harness this potential to enhance responsible antimicrobial use.

In contrast to knowledge and attitudes, the findings of this study revealed poor AMR among HCWs, with an overall good practice score of 43.3%, signifying that a substantial proportion of HCWs exhibited poor antimicrobial use. Conversely, a meta-analysis reported a higher practice level of 54.6% among HCWs in Ethiopia [20], implying a relatively better practice standard within hospital settings compared to health centers. However, in this study, the level of knowledge of HCWs was not sufficient to bring about changes in practices related to AMR at the health centers.

Multivariate analysis revealed several factors influencing HCWs' practices related to AMR. These insights offer valuable guidance for designing targeted interventions. The findings of this study showed that participants aged 20–29 and 30–39 years had significantly poorer practices (81% and 71%, respectively) toward AMR than did those aged 40 or older (AOR: 0.19, 95% CI: 0.08–4.72; AOR: 0.29, 95% CI: 0.12–0.70). Age emerged as a significant factor, with younger HCWs exhibiting poorer practices. However, a study conducted in Egypt and West London showed that there is no statistically significant relationship between age and antimicrobial prescribing practices [28]. This underscores the need for targeted training programs on AMR, antimicrobial stewardship, and evidence-based prescribing, particularly among younger healthcare professionals in the study area.

The profession also played a role in practice differences, with general practitioners demonstrating significantly better practices than nurses. General practitioners performed 4.26 times better in AMR practice than nurses did (AOR: 4.26, 95% CI: 1.52–11.90). However, a study conducted in Sweden revealed that interns and residents prescribed appropriate antibiotics based on the guidelines rather than general practitioners who prescribed antibiotics that differed from the standard guidelines [29]. This finding indicated that all HCWs should follow the standard guidelines when prescribing antibiotics.

Education level played a role in this study, as participants with a degree had 53% lower odds of engaging in antimicrobial prescribing practices than did those with a master's degree (AOR: 0.47, 95% CI: 0.26–0.88). This finding is supported by a meta-analysis of 42 countries with different socioeconomic statuses that revealed that higher education is associated with 14% lower odds of antibiotic misuse in lower- to middle-income countries, in contrast to Europe, where higher education is associated with 25% greater odds of antibiotic misuse [30]. This might be because advanced degrees may provide a broader understanding of healthcare topics and suggest that educational initiatives should be tailored to the specific needs of different healthcare professions for responsible antimicrobial use.

The department of practice within healthcare facilities was another influential factor. The study revealed that HCWs working in the outpatient department exhibited 3.78 times better AMR practices than did inpatient HCWs (AOR: 3.78, 95% CI: 1.82–7.86). In contrast, OPD visits at primary care in northern Vietnam were more likely to be associated with antibiotic prescriptions than visits in any other department [31]. HCWs in the OPD exhibited better practices than did those in the inpatient department. Understanding these variations can guide interventions, potentially through the sharing of best practices between departments and emphasizing the importance of responsible antimicrobial use, especially in inpatient settings where antibiotic use is often more frequent and complex.

Training on AMR within the past year was associated with better practices, highlighting the positive impact of ongoing education. A lack of training on AMR in the past year significantly reduced the likelihood of demonstrating ‘good practices' toward AMR resistance by 84% compared to training (AOR: 0.16, 95% CI: 0.09–0.29). This finding is supported by evidence from five hospitals in London that showed that only 5%–13% of participants stated that their previous antimicrobial education was effective, and 60% of those who experienced secondary care junior doctors reported wanting further education in antibiotic prescribing [32]. This indicates that effective antibiotic prescription needs continuous and updated training for HCWs. Therefore, healthcare facilities should prioritize regular training and ensure that HCWs have access to up-to-date information on AMR and antimicrobial stewardship.

Both knowledge and attitude scores significantly influenced practices. Surprisingly, HCWs with “knowledgeable” knowledge about AMR had 55% lower odds of engaging in “good practices” than did those with “not good knowledge” (AOR: 0.45, 95% CI: 0.28–0.72). This might indicate that antimicrobial prescriptions are mainly driven by empiric decisions, as revealed in another study conducted in tertiary care hospitals in Ethiopia [33]. Positive attitudes toward AMR were associated with greater odds of demonstrating ‘good practices' (AOR: 1.79, 95% CI: 1.13–2.85). Those with a positive attitude toward AMR demonstrated better practices. These findings reinforce the importance of addressing not only knowledge gaps but also attitudes and beliefs in educational interventions.

This study revealed that HCWs who did not adhere to any guidelines before they were prescribed antimicrobials experienced poor practices by 66% (AOR: 0.34, 95% CI: 0.22–0.52). A study conducted in Uganda revealed that 79% of health workers reported following clinical guidelines when prescribing antibiotics [34], and a study in Iran showed that 65.9% of participants in hospitals used local and international antimicrobial therapy guidelines before prescribing antibiotics [35]. This might indicate that adhering to guidelines before prescribing antibiotics improves practice.

### 4.1. Strengths and Limitations of the Study

As this cross-sectional study is reliant on self-reported data, participants may have provided socially desirable responses, particularly regarding practices and attitudes, potentially overestimating adherence to AMR guidelines. The study captures a snapshot of KAP at a single point in time, limiting the ability to infer causality in behaviors and knowledge over time. While focused on primary healthcare centers, the sample may not fully represent all HCW groups or geographic areas within Addis Ababa. The study does not explore systemic barriers or cultural influences that may shape AMR practices.

Despite these limitations, the study provides foundational insights into AMR-related KAP among Ethiopian HCWs, offering a platform for future longitudinal research, mixed-methods approaches, and targeted interventions to combat AMR in resource-limited settings.

## 5. Conclusions and Recommendations

The study highlights critical gaps in AMR practices among HCWs in Addis Ababa, Ethiopia. While HCWs demonstrated good knowledge (66.8%) and unfavorable attitudes (61.3%), their overall practices were notably poor (43.3%). Factors such as age, profession, education, department (inpatient/outpatient), lack of training, and inconsistent adherence to guidelines contribute to suboptimal practices. These findings highlight the urgent need for targeted interventions to bridge the gap between theoretical understanding and practical implementation of AMR mitigation strategies.

To address these challenges, the Addis Ababa City Administration Health Bureau should prioritize evidence-based interventions. HCWs must be equipped with comprehensive knowledge about the mechanisms of AMR, the importance of appropriate antimicrobial use, and the implementation of universal precautions such as hand hygiene, the use of personal protective equipment, and safe handling of infectious materials. A positive attitude toward adhering to these practices is essential, as it promotes a culture of safety and accountability within healthcare settings. Regular training and awareness programs can enhance KAP, ultimately leading to improved patient outcomes and reduced incidence of AMR in healthcare facilities.

## Figures and Tables

**Table 1:** Sociodemographic characteristics of the study participants, Addis Ababa, Ethiopia, September 2023.

Variables	Categories	Frequency	Percentage (%)
Sex	Male	309	65.3
	Female	164	34.7

Age	20–29	252	53.3
	30–39	182	38.5
	≥ 40	39	8.2

Educational level	Diploma	62	13.1
	Degree	348	73.6
	Masters	63	13.3

Profession	General practitioner	27	5.7
	Health officer	183	38.7
	Nurses	263	55.6

Year of experience	≤ 5	206	43.6
	6–10	190	40.2
	11–15	57	12.1
	≥ 16	20	4.2

Ever had training on AMR	Yes	321	67.9
	No	152	32.1

**Table 2:** Factors associated with practices of AMR in Addis Ababa, Ethiopia, September 2023.

Variables	Categories	Practice toward antimicrobial resistance		Crude odds ratio (95% CI)	AOR (95% CI)
		Good	Poor		
Age category	20–29	101 (40)	151 (60)	0.38 (0.19–0.76)	0.19 (0.08–4.72)
	30–39	79 (43.4)	103 (56.6)	0.43 (0.21–0.88)	0.29 (0.12–0.70)^∗^
	≥ 40	25 (64.1)	14 (35.9)	1	1

Profession	GP	19 (70.4)	8 (29.6)	3.36 (1.42–7.94)	4.26 (1.52–11.90)^∗^
	Health officer	77 (42.1)	106 (57.9)	1.03 (0.70–1.51)	0.96 (0.59–1.55)
	Nurse	109 (41.4)	154 (58.6)	1	1

Highest level of education	Diploma	25 (40.3)	37 (59.7)	0.66 (0.32–1.33)	0.62 (0.26–1.50)
	Degree	148 (42.5)	200 (57.4)	0.72 (0.42–1.23)	0.47 (0.26–0.88)^∗^
	Masters	32 (50.7)	31 (49.3)	1	1

Department of practice in the past 6 months	OPD	189 (45.9)	223 (54.1)	2.38 (1.31–4.35)	3.78 (1.82–7.86)^∗^
	IPD	16 (26.2)	45 (73.8)	1	1

Received training related to AMR in the past year	No	135 (36)	240 (64)	0.23 (0.14–0.37)	0.16 (0.09–0.29)^∗^
	Yes	70 (71.4)	28 (28.6)	1	1

Knowledge score	Not good	82 (52.2)	75 (47.8)	1	1
	Good	123 (38.9)	193 (61.1)	0.58 (0.40–0.86)	0.45 (0.28–0.72)^∗^

Attitude score	Unfavorable	63 (34.4)	120 (65.6)	1	1
	Favorable	63 (49)	148 (51)	1.83 (1.25–2.77)	1.79 (1.13–2.85)^∗^

Consulted guidelines before prescribing antimicrobials	No	76	170	2.94 (2.02–4.29)	0.34 (0.22–0.52)^∗^
	Yes	129	98	1	1

^∗^Significant (*p* values < 0.05).

## Data Availability

The data that support the findings of this study are available from the corresponding author upon reasonable request.

## Nomenclature

AMR: Antimicrobial resistance
AMS: Antimicrobial stewardship program
AOR: Adjusted odds ratio
AWaRe: Access, watch, reserve
CI: Confidence interval
COR: Crude odds ratio
HCWs: Healthcare workers
IPC: Infection prevention and control
IPD: Inpatient department
KAP: Knowledge, attitude, and practice
MRSA: Methicillin-resistant *Staphylococcus aureus*
OPD: Outpatient department
SD: Standard deviation
WHO: World Health Organization
